# Context of implementation of mental health fremekork in Blumenau, Brazil (evidence based)

**DOI:** 10.1192/j.eurpsy.2024.1246

**Published:** 2024-08-27

**Authors:** M. A. Cigognini

**Affiliations:** Institute of Neuroscience & Behavior, Blumenau, Brazil

## Abstract

**Introduction:**

Promoting high quality mental health (MS) services is an obligation of many social agents due to the impact of these diseases on the population. Making care increasingly evidence-based does not depend exclusively on technical training, but also on gradual and functional changes in the structure of an institution. Improving the quality of services in MH is a predominantly social intervention, in which it is necessary to group and interpret complex data. They represent real-time interventions in a real world by teams delivering health services.

**Objectives:**

Describe the context where the MH service (iNC) is inserted, its main characteristics and purposes.

**Methods:**

Mixed study identifying the location, socio-demographic data, characteristics and fundamentals of an organization providing services in MH that proposes to act based on evidence.

**Results:**

iNC is a private secondary care institution located in the city of Blumenau, Vale do Itajaí, state of Santa Catarina, Brazil (FIG 1). Vale do Itajaí is a mesoregion with approximately 1.5 million inhabitants composed of small regions: Blumenau, Itajaí Ituporanga and Rio do Sul. Most of the population is of German and Italian descent. Blumenau has 361,261 inhabitants, an average monthly income of 2.9 minimum wages, 97% of schooling between 6-14 years old and the number of deaths of 6.48 (1000 live births). iNC is located in the center of the city (3-story building) with a clinical staff idealized for 3 psychiatrists, 16 psychologists, 4 nutritionists, 1 nurse, 1 nursing technician and 1 physical educator in face-to-face and online, individual and in-person sessions group. Performs care for adult patients between 18-70 years. Its missions are: to promote MS care from an interdisciplinary perspective, to provide health interventions supported by the best individualized scientific evidence and to encourage teaching and research in the field of MH. Its guiding principles are: psychopathology and nosology (DSM-5 and CID-11), neuroscience and psychopharmacology, mood, anxiety, sleep, eating and obesity disorders (FIG 2), psychological treatments and psychoeducation, assessment instruments in MH and neuropsychology, evidence-based medicine, health promotion and disease prevention.

**Conclusions:**

Identifying, measuring and quantifying a local assistance service in MH can help in its development and allow comparisons over time. The improvement of services depends on multiple factors and is necessary for their development, both for researchers, implementers, health professionals and patients.

**Disclosure of Interest:**

None Declared

